# Clinical and psychosocial factors associated with quality of life in patients with head and neck cancer: an analytical cross-sectional study from a lower-middle-income country

**DOI:** 10.1186/s40359-023-01264-6

**Published:** 2023-09-05

**Authors:** Nida Zahid, Russell Seth Martins, Zaiba Shafik Dawood, Wajeeha Zahid, Iqbal Azam, Mubasher Ikram, Aneesa Hassan, Shireen Shehzad Bhamani, Nargis Asad, Adnan Abdul Jabbar, Shabbir Akhtar, Moghira Iqbaluddin Siddiqui, Mohammad Sohail Awan, Khabir Ahmad

**Affiliations:** 1 Department of Surgery, Aga Khan University Hospital, Karachi, Pakistan; 2 Division of Thoracic Surgery, Department of Surgery, JFK University Medical Center, Hackensack Meridian Health, Edison, NJ 08820 United States of America; 3 Medical College, Aga Khan University Hospital, Karachi, Pakistan; 4 Department of Community Health Sciences, Aga Khan University Hospital, Karachi, Pakistan; 5 School of Nursing and Midwifery, Aga Khan University Hospital, Karachi, Pakistan; 6 Department of Psychiatry, Aga Khan University Hospital, Karachi, Pakistan; 7 Department of Oncology, Aga Khan University Hospital, Karachi, Pakistan

**Keywords:** Quality of life, Psychosocial factors, Head and neck cancer, Oncology, Depression, Anxiety, Low- and middle-income country

## Abstract

**Introduction:**

The disease course of head and neck (H&N) cancer can severely impair patients’ quality of life (QoL). In Pakistan, a South Asian lower-middle-income country (LMIC), psychosocial factors may impact QoL. We aimed to assess QoL and associated factors amongst patients with H&N cancer in Pakistan.

**Methods:**

An analytical cross-sectional study was conducted amongst adult (≥ 18 years) patients with H&N cancer who were ≥ 4 weeks post-initiation of treatment. The survey assessed QoL (European Organization for Research and Treatment of Cancer Quality of Life Questionnaires), anxiety and depression (Hospital Anxiety and Depression Scale), and social support (Enriched Social Support Instrument). Multivariable linear regression was performed for analysis.

**Results:**

A total of 250 patients (mean age: 51.6 years) were included. The majority of patients were married (87%) and living with multigenerational/extended family households (53%). On multivariable linear regression, ongoing cancer treatment (beta coefficient: -13.93), having a tracheostomy (-10.02), and receiving adjuvant chemoradiotherapy (-8.17) were significantly associated with poorer global QoL. Additionally, poorer QoL was associated with depression (-24.37) and anxiety (-13.34). However, having more household family members was associated with better global QoL (0.34).

**Conclusion:**

The QoL of patients with H&N cancer in Pakistan is affected by both the nature of cancer treatment as well as sociocultural factors such the number of household family members. Given that poorer QoL is associated with worse mental health outcomes, there is a need to develop and implement psychosocial interventions to improve the QoL of patients with H&N cancer in Pakistan, particularly during active treatment.

## Introduction

Globally, over 900,000 new cases of head and neck (H&N) cancer are diagnosed every year [1], with males being disproportionately affected [2]. Over 500,000 deaths are attributable to H&N cancer annually [1], making it the seventh leading cause of global mortality [2]. In Pakistan, a lower-middle-income country in South Asian, H&N cancers account for over 18% of newly diagnosed cancers, with most cases linked to consumption of tobacco, alcohol, and betel nut [3]. H&N cancers are the second leading cause of cancer-related mortality in the country, after breast cancer [4]. The clinical course of H&N cancer, including its management, causes great distress to patients. Both the disease and its treatment may lead to the loss of body parts, scarring, hair loss, disfigurement, and weight changes, which can affect self-perception and body image [5]. In addition, functional impairment can include problems in speech, swallowing, and social interactions [6, 7]. These adverse consequences can seriously impact patients’ quality of life.

Quality of life (QoL) is a multidimensional construct involving the subjective perceptions of positive and negative aspects of disease symptoms, physical, emotional, social, cognitive functions, and the side effects of treatment [8]. In patients with H&N cancer, QoL is especially important due to reduced physical and social functionality, as well as impairment of the senses of taste and smell [7]. Reductions in QoL occur fairly early in the disease course of H&N cancer and may be further lowered by treatment interventions such as surgery, chemotherapy, and radiotherapy [9, 10]. Even survivors of H&N cancer suffer from lingering QoL impairments due to pain, physical disability, and fatigue [11]. Poorer QoL may be associated with significant adverse mental health outcomes, including anxiety, depression, and general distress [12, 13]. Thus, QoL is an important outcome measure in patients with cancer, as treatment rarely offers complete recovery and life expectancy may frequently be limited [9]. While several tools exist for the measurement of QoL in H&N cancer, the European Organisation for Research and Treatment of Cancer (EORTC) QoL Questionnaire (QLQ30) and its H&N specific module (H&N35) are amongst the most widely used and extensively validated [14].

In Pakistan, the QoL of patients with H&N cancer has been a neglected field of study. The reasons for this are several. Firstly, the country in general lacks established research infrastructure, human resources, and funding. Moreover, very few tools assessing patient-reported outcomes like QoL are available in Urdu, which is Pakistan’s national language. Only a single study of 84 patients has been published, which used only the EORTC QLQ30 to report changes in QoL before and after treatment [15]. Factors associated with QoL amongst patients with H&N cancer remain to be explored. These include socioeconomic factors, which are particularly relevant in the context of a lower-middle-income country (LMIC) like Pakistan [16]. Social support may plan an important role in protecting QoL in patients with cancer [17]. Pakistan’s social framework is different to West, where most of QoL-related literature originates, as it consists predominantly of joint family structures as opposed to the nuclear families seen in the West [18]. Moreover, previous research by our team has demonstrated that patients with cancer in Pakistan have greater resilience than their counterparts in the West [19–21]. We hypothesized that this difference may arise due to perennial exposure to socioeconomic stressors, which may increase baseline resilience amongst the general population. Differences economic and psychosocial characteristics of the population Pakistan invite research exploring associations of these factors with QoL outcomes. Thus, we aimed to explore factors associated with QoL of patients with H&N cancer in Pakistan, with a particular focus on sociodemographic and psychosocial characteristics.

## Methods

### Study design and participants

An analytical cross-sectional survey-based study was conducted from November 2019 to May 2020 among adult (≥ 18 years) patients with head and neck (H&N) cancer at the Aga Khan University Hospital (AKUH), a tertiary care hospital in Pakistan. Patients were included if they were currently ≥ 4 weeks post-initiation of treatment at AKUH, living in Pakistan since the past 3 months, and provided written informed consent for participation in the study.

We excluded patients with debilitating physical ailments (stroke, renal failure) or psychiatric disorders, as these conditions would confound QoL outcomes. Patients with comorbid conditions such as hypertension, cardiovascular diseases (CVD), diabetes mellitus or chronic obstructive pulmonary disease (COPD) were not excluded. Given the high prevalence of these comorbid conditions in the Pakistani population [22], their use as exclusion criteria would hinder sample size achievability and result in a non-representative sample. Therefore, these confounders were adjusted for during analysis [23].

### Survey tool

The survey tool consisted of a structured questionnaire in Urdu, the national language of Pakistan. It included the following sections:

- *Patient Sociodemographic Characteristics*: Patients’ age, gender, ethnicity, education, family and household situation, comorbid conditions (hypertension, diabetes, cardiovascular disease), history of addictions (including smoking, substance abuse), employment status of patient and family members, and monthly household income. These are described in full in a prior study from the same patient cohort [34].
- *Patient Disease-related Characteristics*: Type of tumor, surgery, chemotherapy and/or radiotherapy and site of tumor. These are described in full in a prior study from the same patient cohort [34].
- *Psychosocial Characteristics*: Data regarding depression and anxiety was collected via the validated Urdu version of the Hospital Anxiety and Depression Scale (HADS) which comprises of 14 items equally subdivided into the anxiety and depression subscales with each item scored from 0 to 3 [30, 31]. An example of an item is as follows: “*I feel tense or ‘wound up’*”. Responses include: “3: *Most of the time*; 2: *A lot of the time*; 1: *From time to time/occasionally*; and 0: *Not at all*”. Individuals scoring between 8 and 10 were classified as mildly anxious and depressed, whereas those scoring ≥ 11 were classified as anxious and depressed [22]. The Urdu translation of the HADS [23] has been found to have a good overall Cronbach’s alpha of 0.84 (0.64 and 0.82 for depression and anxiety subscales, respectively) [24]. In our current sample of patients with H&N cancer, the overall Cronbach’s alpha was 0.88, with those of the anxiety and depression subscales being 0.79 and 0.84, respectively. Data regarding social support (functional and emotional) was collected through the validated Urdu version of the ENRICHD Social Support Instrument (ESSI) [32] with a CVI for relevance of 0.95, clarity of 0.97, and Cronbach’s alpha of 0.82 [33]. It comprises of 7 items. The first 6 used a 5-point Likert scale numbered 1 to 5. The 7th item is a yes/no question, scored 4 for yes and 2 for no. Total scores range from 8 to 34. An aggregate score of at most 18 was considered as low social support [32]. An example of an item is as follows: “*Is there someone available to you who shows you love and affection?”*. Responses include: “5: *All of the time*; 4: *Most of the time*; 3: *Some of the time*; 2: *A little of the time*; 1: *None of the time*”. Resilience was measured using the validated Urdu version of Wagnild and Young’s 14-item resilience scale (RS-14), where each item is rated on a 7-point Likert scale (strongly disagree to strongly agree) [25]. The test–retest correlation coefficient of the Urdu version of the RS-14 is 0.49, its Cronbach’s alpha is 0.76, and its concurrent validity is 0.813 [26]. An example of an item is as follows: “*I usually take things in stride*”. The response is on a 7-point Likert scale from 7: *Strongly Agree* to 1: *Strongly Disagree*.
- *Quality of Life*: QoL was measured using the Urdu versions of the EORTC QLQ-C30 and H&N35. The comprehensive validation of the Urdu versions of these tools has been described previously by the authors [27, 28]. The EORTC QLQ-C30 is composed of 9 multi-item scales: 5 functioning scales (physical, role, cognitive, emotional and social), a global QOL scale, and 3 symptom scales (fatigue, pain and nausea/vomiting). In addition, several single item symptom measures are used [26]. It has excellent validity, with a content validity index (CVI) of 0.81 for clarity and 0.95 for relevance, and similarly high internal consistency (Cronbach’s alpha ranging from 0.74 to 0.86 for the various scales) [27]. An example of an item is as follows: “*Were you limited in doing either your work or other daily activities?*”. Responses include: “4: *Very much*; 3: *Quite a bit*; 2: *A little*; 1: *Not at all*”.
  The QLQ-H&N35 is a H&N cancer-specific module supplement which incorporates seven multiple-item scales that assess the symptoms of pain, swallowing ability, senses (taste/smell), speech, social eating, social contact, and sexuality. Also included are six single-item scales, which survey the presence of symptomatic problems associated with teeth, mouth-opening, dry mouth (xerostomia), sticky saliva, coughing, and feeling ill. A high score for a symptom scale represents the presence of a symptom or problem(s) (24). An example of an item is as follows: “*Do you have trouble eating?*”. Responses include: “4: *Very much*; 3: *Quite a bit*; 2: *A little*; 1: *Not at all*”.
  Both the EORTC QLQ-C30 and QLQ-H&N35 scales employ a 4-point response format (“not at all” to “very much”), with the exception of the global QoL scale, which has a 7-point response format. Scale scores are transformed to a scale from 0 to 100 according to the EORTC scoring algorithm (28). For the functioning and the global QoL scale, a higher score indicates better health. For the symptoms scales, a higher score indicates a higher level of symptom burden (26, 29).

Prior to participant recruitment, the questionnaire was piloted on 5% of the sample size to identify any ambiguities. No major changes were deemed necessary after this pilot testing.

### Sample size and sampling strategy

The minimum sample size was calculated to be 250 based on mean QoL scores for head and neck cancer patients from previous studies [24, 25]. It was calculated using the one population mean formula, based on a standard deviation (SD) range of 16.5–40.8, 5% level of significance with precision of 2.5, and adding non-response of 10% [29–31].

Nonprobability purposive sampling technique was employed. Trained research assistants approached all patients with H&N cancer visiting the surgical/oncology clinics at AKUH as per their scheduled appointments. Potential participants were screened for eligibility. Eligible participants were briefed on the scope and nature of the study, as well as the extent of their participation. Patients who provided written informed consent for participation were enrolled in the study, and the study questionnaire was administered to them by the research assistants. The administration of the questionnaire took approximately 30–40 min [29–31].

The details of sample size and sampling strategy are published in previous papers from the same project [29–31].

### Plan of analysis

The data was analyzed on STATA version 15. Continuous variables were reported as mean ± standard deviation (SD)/median (IQR), while categorical variables were reported as frequency and percentages. The dependent variable was global QoL. The independent variables were demographic variables (age, gender, monthly income, working status), schooling background, role in family, medical comorbid conditions (hypertension, diabetes mellitus, CVD), use of addictive substances (tobacco and alcohol), family history of cancer, tumor and treatment-related factors (type of tumor, type of surgical intervention, and adjuvant therapy), social support, depression and anxiety.

General Linear Model (GLM) multivariate analysis of variance (MANOVA) was used to determine the relationship between the independent variables and the dependent variables (QLQ-C30 and QLQ-H&N34 scales and subscales). Initially, a one-factor model was used to identify independent variable with a p < 0.20. These variables were then assessed on a multi-factor model. In the GLM-MANOVA model, age was considered a covariate and all other variables (i.e., all categorical variables) were considered fixed factors.

Linear regression was also used to report unadjusted and adjusted beta coefficients with 95% confidence intervals (CI), to determine the factors associated independently with QoL. A p value < 0.20 on univariate regression was considered the cut-off for inclusion into the multivariable regression model. Throughout all analyses, p < 0.05 was considered significant.

### Ethical considerations

The study was approved by the Ethical Review Committee of the Aga Khan University Hospital. Written informed consent was obtained from all the study participants. Patient information was kept confidential, and no personal identifier was disclosed. Participants identified as having mild-moderate depression or anxiety via the HADS were provided on-the-spot counseling by a trained psychologist. Patients identified as having severe depression or anxiety (or having suicidal thoughts) were referred for urgent psychiatric care.

## Results

### Study participants

The sample comprised of 250 patients with H&N cancer, with the most common type being oral cancer (82%). Their average age was 51.59 years, and most patients (79%) were male. The majority of patients had received a formal education (87%). Urdu was the mother tongue of half of the patients, though all were able to understand and speak Urdu fluently. The majority of patients (87.2%) were married. Just over half (53.6%) lived in extended families and 51.6% of the patients had 6 or more family members in their household. Additionally, 55% of patients were family heads or decision-makers within their households.

Almost two-thirds of patients were unemployed (64%) and 73% had spouses who were unemployed. The median monthly household income was PKR 45,000 (range: PKR 22,650–100,000). Further details of patients’ socio-demographic characteristic, medical history, tumor and treatment related factors, social support and mental health are shown Tables 1, 2 and 3 of the **previous publication** by the authors based on analysis of the same sample [29–31].

**Table 1:** Quality of life (QoL) of patients with H&N cancer as measured by the EORCT QLQ-C30 and QLQ-H&N35

QLQ-C30 Component	Mean (SD)
Global QoL	72.23 (18.48)
**Functional Scale**	
Physical Functioning	78.42 (20.83)
Role Functioning	81.00 (26.69)
Emotional Functioning	79.63 (20.60)
Cognitive Functioning	88.53 (17.94)
Social Functioning	80.20 (27.50)
**Symptom Scales**	
Fatigue	13.93 (19.52)
Nausea	14.33 (16.80)
Pain	61.53 (27.31)
Dyspnea	10.40 (21.43)
Insomnia	30.80 (25.52)
Appetite Loss	34.40 (30.98)
Constipation	5.20 (17.82)
Diarrhea	17.60 (29.54)
Financial Difficulties	33.73 (39.07)
**QLQ-H&N 35 Component**	**Mean (SD)**
**Symptom Scale**	
Pain	13.93 (19.53)
Swallowing	14.33 (16.80)
Senses	61.53 (27.31)
Speech	32.31 (33.71)
Trouble eating	30.76 (25.46)
Trouble contacting	14.88 (23.01)
Less sexuality	44.77 (37.07)
Teeth	20.80 (34.61)
Opening mouth	46.00 (38.68)
Dry Mouth	40.26 (34.31)
Sticky Saliva	20.53 (35.32)
Cough	24.80 (30.33)
Felt ill	25.86 (29.22)
Pain killers	70.00 (45.90)
Nutritional supplements	71.60 (45.18)
Feeding tube	49.60 (50.09)
Weight loss	55.60 (49.78)
Weight gain	73.20(44.38)

**Table 2:** Association of clinicodemographic variables with QLQ-C30 and QLQ-H&N35 using GLM-MANOVA. GLM-MANOVA: General Linear Model Multivariate Analysis of Variance. * p value < 0.2 by one factor model of GLM-MANOVA. ** p value < 0.05 by multifactor model of GLM-MANOVA

EORTC QLQ-C30		
Variables	One factor Model Wilk λ (p value)	Multifactor Model Wilk λ (p value)
Age (in years)	0.768 *	0.835 (< 0.001)**
Working status	0.805*	0.796 (< 0.001)**
Household Monthly Income (in PKR)	0.499*	0.555 (< 0.001)**
Hypertension	0.911*	NS
Diabetes Mellitus	0.915*	NS
Smokeless tobacco use	0.746*	0.783 (0.004)**
Family history of cancer	0.916*	NS
Types of tumor	0.798*	NS
Treatment of cancer	0.786*	NS
Types of Intervention	0.663*	0.71 (0.002)**
Feeding tube	0.73*	NS
Urine bag	0.841*	NS
Resilience	0.627*	0.824 (0.0001)**
Depression	0.459*	0.65 (< 0.001)**
Anxiety	0.649*	0.721 (< 0.001)**
Social support	0.854*	0.892 (0.041)**
**EORTC QLQ H&N 35**		
**Variables**	**One factor Model** Wilk λ (p value)	**Multifactor Model** Wilk λ (p value)
Age (years)	0.851*	0.855 (0.0129)**
Gender	0.864*	0.825(0.0018)**
Monthly household income (PKR)	0.555*	0.599(0.003)**
Working status	0.831*	0.732 (< 0.001)**
Hypertension	0.886*	NS
Diabetes Mellitus	0.885*	NS
Smoking	0.772*	NS
Smokeless tobacco use	0.759*	NS
Family history of any other cancer	0.853*	NS
Types of tumor	0.682*	NS
Treatment of cancer	0.577*	NS
Types of Intervention	0.577*	NS
Adjuvant therapy	0.691*	0.678 (0.006)**
Urine bag	0.891*	NS
Resilience	0.562*	0.739 (< 0.001)**
Depression	0.739*	0.578 (< 0.001)**
Anxiety	0.482*	NS
Social support	0.775*	0.811(< 0.001)**

**Table 3:** Univariate and multivariable linear regression with Global QoL as dependent variable. SE: standard error; CI: confidence interval; NS non-significant. * p < 0.20 on univariate analysis. ** p < 0.05

Variables	Univariate Analysis	Multivariable Analysis
	Beta Coefficient (SE) [95% CI]	Beta Coefficient (SE) [95% CI]
Formal Schooling		
Yes (Reference)	Reference	NS
No	-5.18 (3.47) [-12.03, -1.65] *	
Number of people in the household	0.32 (0.15) [0.007, 0.632] *	0.34 (0.14) [0.06, 0.63] **
Monthly Family Income (PKR/USD)		
No Income	− 10.32 (4.85) [-19.88, -0.76] *	
6000–25,000/ 38–151	-15.14 (3.06) [-21.17, -9.09] *	
25,000–40,000/151–242	-4.41 (3.47) [-11.26, 2.44] *	
40,000–80,000/242–484	-5.74 (3.03) [-11.72,0.24] *	
80000-1500000/484–1028	Reference	NS
Hypertension	-4.41 (2.65) [-0.81, 9.64] *	NS
Status of Cancer Treatment		
Complete (Reference)	Reference	Reference
Ongoing	-13.32 (2.19) [-17.65, -9.01] *	-13.93 (2.16) [-18.20, -9.66) **
Feeding Tube		
No (reference)	Reference	
Yes	-11.86 (2.24) [-16.27, -7.45] *	NS
Tracheostomy		
No(reference)	Reference	
Yes	-14.09 (4.30) [22.57, -5.61] *	-10.02(3.94) [2.24, 17.80] **
Using Urine Catheter		
No (reference)	Reference	
Yes	-22.85 (6.59) [-35.85, -9.87] *	NS
Adjuvant Therapy		
No Adjuvant therapy Chemotherapy Radiotherapy Combination	Reference 1.66(5.74) [-12.98, 9.64] 3.13(3.56) [-3.87, 10.14] -5.39 (2.82) [ -10.96, 0.179]	Reference -4.36(5.23) [-14.67, 5.94] -2.54(3.30) [-9.05, 3.96] -8.17(2.62) [-13.36, -2.99] **
Resilience	0.83 (0.10) [0.64, 1.03] *	NS
Social Support		
≤ 18 (Low Social Support)	-12.48 (4.43) [-21.22, -3.74] *	
> 18 (High Social Support)	Reference	NS
Depression		
0–7 (Normal) 8–10 (Mild Depression) 11–21 (Symptomatic Depression)	Reference -13.36 (4.43) [-21.22, -3.74] * -29.71 (3.94) [-37.49, -21.97] *	Reference -6.64 (4.22) [-14.96, 1.68] -24.37 (2.48) [-32.42, -16.32] **
Anxiety		
0–7 (Normal) 8–10 (Mild Anxiety) 11–21 (Symptomatic Anxiety)	Reference -18.34 (6.93) [-31.98, -4.68] * -14.76 (7.46) [-29.48, -0.05] *	Reference -13.34 (5.88) [-24.92, -1.76] ** 0.85 (6.93) [-12.80, 14.50]

### Quality of life in patients with H&N cancer

The mean global QoL score was 72.23. The mean scores of the QLQ-C30 functioning scales ranged from 88.53 (cognitive functioning) to 78.42 (physical functioning). In the QLQ-C30 symptom scales, pain had the highest score (61.53), followed by appetite loss (34.40), financial difficulty (33.73), and insomnia (30.80). The worst symptoms on the QLQ-H&N35 symptom scale were weight gain (73.20) and pain killers (70.00). These are detailed in Table 1.

### Factors associated with QoL outcomes among head and neck cancer patients

A total of 16 variables were included in the multifactor model GLM-MANOVA with EORTC QLQ-C30 scales as the dependent variables (Table 2). Variables significantly associated on the multifactor model were age, working status, household monthly income, use of smokeless tobacco, type of intervention, resilience, depression, anxiety and social support (all p < 0.05).

A total of 18 variables were included in the multifactor model GLM-MANOVA with EORTC QLQ-H&N35 scales as the dependent variables (Table 2). Variables significantly associated on the multifactor model were age, working status, household monthly income, use of smokeless tobacco, type of intervention, resilience, depression, anxiety and social support (p < 0.05).

### Linear regression analysis

On multivariable linear regression (Table 3), ongoing cancer treatment (beta coefficient: -13.93), having a tracheostomy (-10.02), and adjuvant chemoradiotherapy (-8.17) were significantly associated with poorer global QoL. Additionally, symptomatic depression (-24.37) and mild anxiety (-13.34) were also associated with poorer QoL. However, having more household family members was associated with better global QoL (0.34).

## Discussion

The influence of sociodemographic and psychosocial characteristics on the QoL of patients with H&N cancer is poorly understood in the context of a lower-middle-income country like Pakistan. Our study, the first of its kind, used the EORTC QLQ-C30 and H&N35 to investigate factors associated with the QoL of patients with H&N cancer in Pakistan. Our results showed ongoing cancer treatment, presence of a tracheostomy, and receiving combination adjuvant therapy to be independently associated with poorer global QoL. Amongst psychosocial characteristics, depression and anxiety were associated with poorer QoL, whereas having more household family members was associated with better QoL.

Patients with H&N cancer in our study had better global QoL (average: 72.23) compared to cohorts in Spain (33.32–70.81) [32], China (54.60) [33], and India (14.12–34.82) [34]. The scores on the EORTC QLQ-C30 general symptom scales were also lower (more favorable) that those seen in the study from Spain [32], but comparable to studies from China [33], Korea [35], and Brazil [36]. Although the multifaceted nature of QoL makes it hard to identify a single reason for the better global QoL seen in our study, it is possible that psychosocial differences in Pakistan may play a role. Our results show that more household family members were associated with better QoL. The extended family structure common to Pakistan [18] may provide much needed day-to-day physical and emotional support to a patient navigating the demanding clinical course of H&N cancer [37]. Interestingly, while social support was associated with QoL on the GLM-MANOVA multifactor models, high social support did not demonstrate an independent protective effect for patients’ QoL on adjusted multivariable regression. This suggests that it is the closer-to-home, familial relationships that nurture QoL [38]. Similarly, while monthly household income was associated with QoL on the GLM-MANOVA multifactor models, it did not retain significance on adjusted multivariable regression.

Though the lack of proper assessment of mental health conditions has been linked to poorer QoL in patients with H&N cancer [39], such assessment is far from being routine practice in Pakistan. However, while depression and anxiety were associated with poorer QoL in our study, the prevalence of these mental health conditions (< 15% of patients with any depression and < 6% of patients with any anxiety) was low. Previous studies employing the HADS in patients with H&N cancer have varying rates of anxiety (12–44%) and depression (10–58%) at varying timepoints during the clinical course of the disease and its treatment [40]. Our cohort demonstrated lower rates of anxiety and depression compared to most previously studied populations, with our results being most similar to a study from another South Asian lower-middle-income country, India [40]. Once again, it is possible that these more favorable mental health conditions may be due to the unique extended family structures seen in South Asian countries such as Pakistan and India. Additionally, more than two-thirds of patients in our cohort were found to have moderate-high resilience, which is a known protective factor for both mental health, including anxiety and depression, amongst patients with cancer [41]. However, other nuanced mechanisms may exist to explain the lower rates of adverse mental outcomes seen in our study, and this warrants future exploration. Nevertheless, despite the relatively low rates of psychological comorbidity seen in our cohort, the routine assessment of mental health conditions should be prioritized in the management of patients with H&N cancer in Pakistan. Both anxiety and depression tend to worsen with time over the course of H&N cancer treatment [42], with consequent impairment of QoL [43]. Lastly, while resilience was significantly associated with QoL on the GLM-MANOVA multifactor models, it did not remain significantly protective of global QoL on adjusted multivariable regression.

The H&N cancer-specific symptoms, such as dry mouth, mouth opening, and loss of senses (taste, smell), were significantly worse in our study compared to those seen in studies from China [33], Brazil [36], and the US [44]. This could possibly be explained by the fact that 80% of patients in our cohort had oral cancers, which cause more severe mouth-related symptomatology, compared to studies in Brazil [36] and the US [44] where cancers of the pharynx and larynx were relatively more common. Additionally, all patients in our study were already on treatment, unlike the study in the US [44] where the cohort included patients who were yet to begin treatment, which could help explain the greater losses of taste and smell in our study. This notion is supported by the findings of our study, which found ongoing cancer treatment (beta coefficient: -13.93), adjuvant chemoradiotherapy (-8.17), and tracheostomy (-10.02) to be significantly and independently associated with lower QoL. Surgery with chemoradiotherapy is the most debilitating treatment for patients with H&N cancer [9] and is understandably associated with poorer QoL due to severe locoregional and systemic side effects incurred by these aggressive therapies. In addition, tracheostomies, due especially to their impairment of speech and social communication, have previously also been shown to lower QoL [45, 46]. The QoL in patients with H&N cancer decreases post-initiation of treatment, remains low during the course of therapy, and even for a short time after completion of treatment [47], emphasizing that this is a critical phase during which efforts should be made to protect QoL. It is only as late as three months post-completion of treatment that QoL begins to increase again [47]. Around this time, the patient is usually better adjusted to the psychological challenges of a cancer diagnosis and has begun to recover from the physical symptomatology incurred during the therapeutic process. Thus, it seems that with regards to QoL of patients being treated for H&N cancer, things tend to get worse before they get better.

### Limitations

Our study has a few limitations that we would like to acknowledge. Firstly, due to the cross-sectional nature of the study, we were unable to investigate changes in QoL during the disease and management course of H&N cancer. Secondly, the majority of patients in our sample were male, which may limit the generalizability our results to female patients in Pakistan. In addition, we were not able to establish a temporal relationship between QoL and mental health outcomes. Additionally, our results are based off results from a single center in Pakistan. However, given that AKUH is the largest private tertiary care hospital in the megacity of Karachi and the patient mix consisted of diverse ethnicities and socioeconomic groups, our results may be reasonably generalizable to other centers in Pakistan.

## Conclusion

This is the first study exploring clinical and psychosocial factors associated with QoL in patients with H&N cancer in Pakistan, a lower-middle-income country in South Asia. The QoL of patients with H&N cancer in Pakistan is affected by both the nature of cancer treatment as well as sociocultural factors. QoL is significantly worse amongst patients currently undergoing treatment, particularly amongst those receiving adjuvant chemoradiotherapy and those needing a tracheostomy. Having more family members is protective of QoL. However, poorer QoL is associated with worse mental health outcomes. Thus, there is a need to develop and implement psychosocial interventions to improve the QoL of patients with H&N cancer in Pakistan. Future studies must focus on the creation and evaluation of suitable psychosocial interventions to promote QoL of patients with H&N cancer in the active treatment phase and beyond.

## Data Availability

The data that support the findings of this study are available from the corresponding author upon reasonable request.

## Declarations

### Original publication

This manuscript has not been submitted or is not under consideration for publication elsewhere. The protocol of the study is published in BMJ Open (doi:10.1136/bmjopen-2019-029084).
